# Assessment of cardiometabolic risk among shift workers in Hungary

**DOI:** 10.1186/1477-7525-10-18

**Published:** 2012-02-01

**Authors:** György Jermendy, Judit Nádas, Ilona Hegyi, István Vasas, Tibor Hidvégi

**Affiliations:** 1 Teaching Department of Medicine, Bajcsy-Zsilinszky Hospital, Maglódi út 89-91, Budapest, 1106, Hungary; 2 Kardirex Medical Center, Táncsics út 43, Győr, 9024, Hungary; 3 Department of Medicine, Petz Teaching Hospital, Vasvári út 2-4, Győr, 9024, Hungary

**Keywords:** Cardiometabolic risk, Metabolic syndrome, Cardiovascular diseases, Circadian rhythm, Shift workers

## Abstract

**Aim:**

Shift workers may be at risk of different diseases. In order to assess cardiometabolic risk in shift workers, a cross-sectional study was performed among active workers.

**Methods:**

A total of 481 workers (121 men, 360 women) were investigated; most of them were employees in light industry (58.2%) or in public services (23.9%). Past medical history was recorded and physical examination was performed. Questionnaires were used to characterize daily activity. Fasting venous blood sample was collected for measuring laboratory parameters. Data from shift workers (n = 234, age: 43.9 ± 8.1 years) were compared to those of daytime workers (n = 247, age: 42.8 ± 8.5 years), men and women were analyzed separately.

**Results:**

In men, systolic blood pressure was higher in shift workers compared to daytime workers (133 ± 8 *vs *126 ± 17 mmHg; p < 0.05). In women, weight (73.6 ± 15.5 *vs *67.7 ± 13.2 kg; p < 0.001), body mass index (27.5 ± 5.7 vs 25.0 ± 4.3 kg/m2; p<0.001) and the prevalence rate of hypertension in the past medical history (24.4 *vs *13.4%; p < 0.01) were higher in shift workers compared to daytime workers. In addition, the proportion of current smokers was higher (37.7 *vs *21.7%; p < 0.001) and HDL-cholesterol level was lower (1.56 ± 0.32 *vs *1.68 ± 0.36 mmol/l; p < 0.01) in female shift workers than in female daytime workers. Both in men and in women, rotating shift workers spent less time sleeping both on working days and on non-working days, spent less time with sport activity, drank more coffee and they spent less time working per day, especially in light physical work, compared to daytime workers. In addition, low and middle educational levels were most frequently found among rotating shift workers as opposed to the daytime workers where high educational level was more common.

**Conclusion:**

Middle-aged active shift workers, especially women, have a less healthy lifestyle and are at higher cardiometabolic risk as compared to daytime workers. Our study highlights the importance of measures for identifying and preventing cardiometabolic risk factors in shift workers.

## Background

It is well known that several physiological functions follow a circadian rhythm, and it is also documented that long-term disturbance of the circadian rhythm have clinical consequences. Obviously, the circadian rhythm of long time shift workers may become disturbed. Accordingly, higher incidence of somatic diseases such as coronary heart disease [1], metabolic syndrome [2] as well as mental and behavioural disorders [3] or sleep disturbances [4] has been described among shift workers.

Cardiovascular risk factors are taken into account in a wider aspect in the concept of cardiometabolic risk than in the metabolic syndrome [5,6]. The concept of cardiometabolic risk has become widely accepted in the literature after the debate on the metabolic syndrome [7]. In addition to waist circumference, blood pressure, HDL-cholesterol, serum triglycerides and fasting blood glucose value, cardiometabolic risk include not only some other classical risk factors (age, gender, ethnicity, physical activity, smoking, LDL-cholesterol) but newer risk factors, such as CRP, as well. The identification of asymptomatic individuals with increased cardiometabolic risk has a great importance because it provides the basis of the primary prevention of cardiovascular diseases. Obviously, early detection of the cardiometabolic risk factors can be achieved only by using screening methods among asymptomatic individuals.

In the present clinical study we examined the occurrence of cardiometabolic risk factors among active shift workers. The screening data of rotating shift (day/night) workers were compared to those of daytime workers.

## Patients and methods

Workers registered in factory medical services were examined in the city of Győr, Hungary. Active workers between the ages of 25-66 years (n = 481, men: 121, women: 360) participated in the screening, 247 (men: 67, women: 180) worked in only daytime and 234 (men: 54, women: 180) worked in rotating (day/night) shift. Employment for at least 5 years under the same daytime or day/night shift pattern was an inclusion criterion. The workers' distribution by workplace was as follows: light industry 58.2%, public service 23.9%, car industry 6.0%, health care 1.0%, transportation 0.4%, food industry 0.4% and other 10.1%.

The screening was performed on a voluntary basis following preliminary information about the study and a date selection. Using pre-printed questionnaires major events of medical history were recorded. In this way, diabetes, hypertension, cardiovascular diseases and abnormal lipids in the past medical history but not earlier than the beginning of the employment were registered. In addition, information on the nature of work (mental, light physical, heavy physical work), on the time spent with work and sleep, on sport activities, smoking, coffee consumption and level of education (low: primary school, middle: secondary school, high: university) was collected. Anthropometric data were recorded (weight, height, waist circumference), body mass index was calculated and blood pressure was measured by standard methods using mercury sphygmomanometers. This was followed by venous blood collection in fasting state; plasma glucose, LDL-cholesterol, HDL-cholesterol, triglycerides, serum creatinine and uric acid values were determined by standard laboratory methods. The measurements were performed on the day of the blood collection and at the same laboratory.

The study was conducted after obtaining the ethical approval from the ETT TUKEB (84-30/2007-1018EKU). All subjects gave their written informed consent prior to participation. The results of their screening were made available for the study subjects accompanied by medical advice if it was necessary.

During the statistical analysis for group comparisons for continuous variables the Mann-Whitney test, for categorical variables the Fisher's exact test were used. Data from men and women were analyzed separately; results of combined analysis (men + women) were not used for characterizing life style and working patterns. The data are presented as mean ± standard deviation (x ± SD) or percentage (%). The level of significance was set at p < 0.05.

## Results

Mostly middle-aged subjects participated in the screening (mean age: 43.4 ± 8.3 years, range: 25-66 years). There was no significant difference between the age of rotating shift workers and daytime workers (43.9 ± 8.1 *vs *42.8 ± 8.5 years) and no significant difference was observed between the age of men and women (42.4 ± 9.1 *vs *43.7 ± 8.1 years). Both men and women have worked for several years (15.6 ± 10.5 and 15.9 ± 10.4 years, respectively) without changes their working place and working patterns (daytime or day/night shift work).

In men, rotating shift workers spent less time sleeping both on working days and on non-working days, spent less time with sport activity, drank more coffee and they spent less time working per day, especially in light or heavy physical work, compared to daytime workers. Within the rotating male shift workers most frequently low and middle educational levels were found as opposed to the daytime workers where high educational level was more common (Table 1). As for the cardiometabolic risk factors, systolic blood pressure proved to be higher in male shift workers compared to daytime workers (Table 2).

**Table 1:** Daily activity and education levels in rotating (day/night) shift workers and daytime workers (men and women analyzed separately)

	Men (n = 121)		Women (n = 360)	
	
	Shift workers (n = 54)	Daytime workers (n = 67)	Shift workers (n = 180)	Daytime workers (n = 180)
Daily worktime (hours)	8.44 ± 1.42**	8.67 ± 1.29	8.22 ± 0.99**	8.25 ± 1.10
Duration of employment (years)	14.3 ± 7.2	16.6 ± 12.6	12.8 ± 7.9***	19.0 ± 11.6
Feature of work (%) mental/light physical/heavy physical	1.8/84.9/13.3***	80.6/19.4/0	0.6/97.7/1.7***	77.6/22.4/0
Duration of sleeping in a workday (hours)	6.3 ± 0.9**	6.9 ± 0.9	6.2 ± 0.9***	7.0 ± 1.1
Duration of sleeping in a non-working day (hours)	7.6 ± 1.8**	8.0 ± 1.0	7.6 ± 1.1***	8.1 ± 1.3
Sport activity yes/no (%)	31.4/68.6	46.3/53.7	17.3/82.7***	35.9/64.1
Weekly sport activity (min)	44.0 ± 95.6*	75.6 ± 102.8	21.9 ± 66.6***	40.0 ± 74.4
Smokers (%) never/ex smokers/current smokers	57.5/16.6/25.9	53.8/32.8/13.4	46.1/16.1/37.8***	66.4/11.7/21.7
Coffee consumption (dose/day)	1.9 ± 1.3**	1.1 ± 1.1	1.9 ± 1.2**	1.6 ± 1.3
Education level (%) low/middle/high	12.9/85.2/1.9***	1.4/34.4/64.2	22.9/76.6/0.5***	2.7/45.6/51.7

*p < 0.05 **p < 0.01 ***p < 0.001 (shift workers versus daytime workers; men and women analyzed separately)

**Table 2:** Clinical and laboratory findings in rotating (day/night) shift workers and daytime workers (men and women analyzed separately)

	Men (n = 121)		Women (n = 360)	
	
	Shift workers (n = 54)	Daytime workers (n = 67)	Shift workers (n = 180)	Daytime workers (n = 180)
Age (years)	42.2 ± 8.1	42.5 ± 9.8	44.5 ± 8.1	42.9 ± 8.0
Weight (kg)	86.5 ± 14.1	90.4 ± 17.6	73.6 ± 15.5***	67.7 ± 13.2
BMI (kg/m^2^)	27.3 ± 3.9	28.7 ± 5.5	27.5 ± 5.7***	25.0 ± 4.3
Waist circumference (cm)	96.9 ± 10.9	101.1 ± 13.2	92.0 ± 13.5	89.2 ± 11.9
Systolic blood pressure (mmHg)	133 ± 18*	126 ± 17	121 ± 19	117 ± 15
Diastolic blood pressure (mmHg)	79 ± 10	79 ± 11	75 ± 11	74 ± 10
Diabetes in the past medical history (%)	3.7	1.4	4.7	1.1
Hypertension in the past medical history (%)	14.4	23.8	24.4**	13.4
CV diseases in the past medical history (%)	1.8	0.0	4.4	1.1
Abnormal lipids in the past medical history (%)	5.5	7.4	7.2	6.1
Fasting blood glucose (mmol/l)	4.87 ± 1.39	4.91 ± 1.16	4.73 ± 0.89	4.56 ± 0.60
Serum LDL-cholesterol (mmol/l)	3.42 ± 0.88	3.70 ± 0.79	3.50 ± 1.01	3.46 ± 0.84
Serum triglycerides (mmol/l)	1.87 ± 1.33	2.18 ± 1.60	1.28 ± 0.69	1.30 ± 0.68
Serum uric acid (μmol/l)	343 ± 66	336 ± 70	265 ± 69	262 ± 57
Serum HDL-cholesterol (mmol/l)	1.34 ± 0.36	1.31 ± 0.35	1.56 ± 0.32**	1.68 ± 0.36
Serum creatinine (μmol/l)	83 ± 10	83 ± 11	66 ± 9	65 ± 9

*p < 0.05 **p < 0.01 ***p < 0.001 (shift workers versus daytime workers; men and women analyzed separately), BMI: body mass index, CV: cardiovascular

In women, the duration of the employment was shorter in shift workers than that in daytime workers. Rotating female shift workers spent less time sleeping both on working days and on non-working days, spent less time with sport activity, drank more coffee, more were current smokers and they spent less time working per day, especially in light physical work, compared to daytime workers. Within the rotating female shift workers most frequently low and middle educational levels were found as opposed to the daytime workers where high educational level was more common (Table 1). Body weight, body mass index were found to be higher within the rotating shift female workers and hypertension was more common than within the daytime workers. From the laboratory data, the HDL-cholesterol level was lower in women with rotating shifts than that of daytime workers (Table 2).

## Discussion

Our data suggest that workers, especially women, in long-term rotating (day/night) shifts have a less healthy lifestyle and a less favourable cardiometabolic risk profile compared to daytime workers.

Several cross-sectional studies have shown that the cardiovascular risk profile of rotating shift (day/night) workers is disadvantageous compared to daytime workers. This could be consistently observed through a range of different employments (police officers, road builders, factory workers, nurses) [8-11]. In a recently published meta-analysis the relationship between rotating shift work and the incidence of ischemic heart disease was examined and indicators of morbidity rather than mortality data were found to be influenced by rotating shift work [12]. Some studies have analyzed the prevalence rate of the metabolic syndrome, and an increase was observed among rotating shift (*vs*. daytime) workers [13,14]. Others have reported on the more frequent occurrence of the individual components of the metabolic syndrome (hypertension, diabetes, lipid abnormalities, obesity) [9,10,15-18]. The results of the cross-sectional observations have also been confirmed by recent follow-up studies [11,19]. Our data - the first observation from Hungary - are consistent with the results of studies published previously.

Although our study cannot be considered representative, the results should be frustrating, especially if one takes into account how many employees may work in shifts. Reliable national statistics are not available in this area, but according to data from industrial countries approximately 20% of the active employees work in rotating or nightshifts [20].

**Figure 1:**
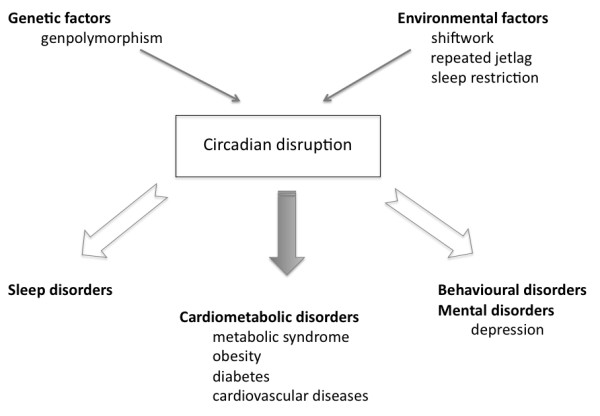
**Causes and possible consequences of circadian rhythm disturbance**.

Our cross-sectional study could not provide data about the possible reasons for the increased cardiometabolic risk of shift workers. According to the generally accepted theory the human circadian biological rhythm is controlled by the neural network of the suprachiasmatic nuclei of the hypothalamus [21]. Certain polymorphisms of the nuclear genes involved in the regulation (CLOCK: circadian locomotor output cycles kaput, BMAL1/ARNTL: brain and muscle aryl-hydrocarbon receptor nuclear translocator-like 1/aryl-hydrocarbon receptor nuclear translocator-like, NPAS2: neuronal PAS domain protein 2) have been shown to be associated with increased body weight and the disruption of circadian rhythm. However, the physiological circadian rhythm can also be damaged due to external factors, which include long-term rotating shift work (21). Due to the disturbance of the circadian rhythm mood disorders (depression), sleep disorders and also an increase in cardiometabolic risk may develop [22,23] (Figure 1). Recent data suggest that melatonin also has a regulatory role [24], as it has been demonstrated that the presence of one of its gene polymorphisms (MTNR1b) increases the risk of developing type 2 diabetes mellitus [25].

In our cohort the rotating shift workers (compared to daytime workers) slept less, both on working days and on non-working days. We do not have data on the quality of sleep. It is typical of industrial countries that in recent years sleep duration has become shorter. More than one-third of the adult population in the United States sleep less than 6 hours per night, and over the past 40 years daily sleeping time has decreased by nearly 2 hours [26]. Sleep deprivation, according to a clinical study, has resulted in an increase in fasting blood glucose and a decrease in insulin sensitivity [27]. During sleep deprivation serum leptin levels were found to be lower, ghrelin levels higher, appetite and consumption of carbohydrate containing foods increased [28]. Another study found increased resistin levels in shift workers [29]. All these alterations can lead to an increase in body weight.

Sleep deprivation and the disruption of circadian rhythm may result in an increase in blood pressure [30] and in a higher incidence of type 2 diabetes mellitus [31] and cardiovascular diseases [32]. A meta-analysis showed that rotating shift work increased the risk of cardiovascular disease by 40% [33]. The increase in body weight is a common phenomenon among rotating shift workers [34]. Data suggestive of these facts have also been provided by our current study. More frequent smoking and less time spent with physical activity among rotating shift workers found in our study are also in accordance with results from other investigations [12].

Among the rotating shift workers most frequently low and middle education levels were found, while in the daytime worker group high education levels were more prevalent. The lower level of education is associated with a more frequent prevalence of the metabolic syndrome or its individual components [35]. This may play a role in the unfavourable cardiometabolic risk profile of shift workers compared to that of daytime workers.

Our study has some limitations. The study design was cross-sectional in nature. The investigation was carried out on a voluntary basis following preliminary information about the study. Participants worked in different working place but mainly in light industry; subgroup analysis according to working places could not be performed due to sample size. Consequently, differences between white-collar and blue-collar workers could not be evaluated. Bearing in mind all these limitations, our data - the first observation from Hungary - should be of interest. It is obvious that recognition and treatment of cardiovascular risk is an important area of the primary and secondary medical care. Appropriate screening for identification of individuals at risk may become the basis of the implementation of primary prevention.

## Conclusion

Our data indicate that middle-aged active shift workers, especially women, have a less healthy lifestyle and are at higher cardiometabolic risk as compared to daytime workers. Therefore, measures for identifying and preventing cardiometabolic risk factors in shift workers are of great importance.

## Competing interests

The authors declare that they have no competing interests.

## Authors' contributions

GJ conceived of the study, participated in its design and coordination, supervised the statistical analysis and wrote the final manuscript. NJ, IH, IV participated in data collection. TH participated in designing and coordinating the study and revised the manuscript. All authors read and approved the finale manuscript.
